# The cost-effectiveness of screening for ovarian cancer: results from the UK Collaborative Trial of Ovarian Cancer Screening (UKCTOCS)

**DOI:** 10.1038/bjc.2017.222

**Published:** 2017-07-25

**Authors:** Usha Menon, Alistair J McGuire, Maria Raikou, Andy Ryan, Susan K Davies, Matthew Burnell, Aleksandra Gentry-Maharaj, Jatinderpal K Kalsi, Naveena Singh, Nazar N Amso, Derek Cruickshank, Stephen Dobbs, Keith Godfrey, Jonathan Herod, Simon Leeson, Tim Mould, John Murdoch, David Oram, Ian Scott, Mourad W Seif, Karin Williamson, Robert Woolas, Lesley Fallowfield, Stuart Campbell, Steven J Skates, Mahesh Parmar, Ian J Jacobs

**Affiliations:** 1 Department of Women’s Cancer, Institute for Women’s Health, University College London, London W1T 7DN, UK; 2 LSE Health & Department of Social Policy, London School of Economics, London WC2A 2AE, UK; 3 Department of Economics, University of Pireaus, Athens GR 18534, Greece; 4 Department of Gynaecological Oncology, Nottingham City Hospital, Nottingham NG5 1PB, UK; 5 Obstetrics and Gynaecology, School of Medicine, College of Biomedical and Life Sciences, Cardiff University, Cardiff CF14 4XN, UK; 6 Department of Gynaecological Oncology, James Cook University Hospital, Middlesbrough TS4 3BW, UK; 7 Department of Gynaecological Oncology, Belfast City Hospital, Belfast BT9 7AB, UK; 8 Northern Gynaecological Oncology Centre, Queen Elizabeth Hospital, Gateshead NE9 6SX, UK; 9 Department of Gynaecological Oncology, Llandudno Hospital, North Wales LL30 1LB, UK; 10 Department of Gynaecological Oncology, Royal Free, London NW3 2QG, UK; 11 Department of Gynaecological Oncology, St. Michael’s Hospital, Bristol BS2 8EG, UK; 12 Department of Gynaecological Oncology, St Bartholomew’s Hospital, London EC1A 7BE, UK; 13 Department of Gynaecological Oncology, Royal Derby Hospital, Derby DE22 3NE, UK; 14 CMFT, St Mary’s Hospital, Manchester M13 9WL, UK; 15 Institute of Cancer Sciences, The University of Manchester, Manchester M13 9PL, UK; 16 Department of Gynaecological Oncology, Nottingham City Hospital, Nottingham NG5 1PB, UK; 17 Department of Gynaecological Oncology, Queen Alexandra Hospital, Portsmouth PO6 3LY, UK; 18 Sussex Health Outcomes Research & Education in Cancer (SHORE-C), Brighton & Sussex Medical School, University of Sussex, Falmer BN1 9RX, UK; 19 Create Health Clinic, London W1G 6AJ, UK; 20 MGH Biostatistics, Massachusetts General Hospital, Boston, MA 02114, USA; 21 Harvard Medical School, Boston, MA 02115, USA; 22 Medical Research Council Clinical Trials Unit at University College London, London WC2B 6NH, UK

**Keywords:** ovarian cancer screening, UKCTOCS, cost-effectiveness, randomised controlled trial, CA125, TVS

## Abstract

**Background:**

To assess the within-trial cost-effectiveness of an NHS ovarian cancer screening (OCS) programme using data from UKCTOCS and extrapolate results based on average life expectancy.

**Methods:**

Within-trial economic evaluation of no screening (C) *vs* either (1) an annual OCS programme using transvaginal ultrasound (USS) or (2) an annual ovarian cancer multimodal screening programme with serum CA125 interpreted using a risk algorithm (ROCA) and transvaginal ultrasound as a second-line test (MMS), plus comparison of lifetime extrapolation of the no screening arm and the MMS programme using both a predictive and a Markov model.

**Results:**

Using a CA125–ROCA cost of £20, the within-trial results show USS to be strictly dominated by MMS, with the MMS *vs* C comparison returning an incremental cost-effectiveness ratio (ICER) of £91 452 per life year gained (LYG). If the CA125–ROCA unit cost is reduced to £15, the ICER becomes £77 818 per LYG. Predictive extrapolation over the expected lifetime of the UKCTOCS women returns an ICER of £30 033 per LYG, while Markov modelling produces an ICER of £46 922 per QALY.

**Conclusion:**

Analysis suggests that, after accounting for the lead time required to establish full mortality benefits, a national OCS programme based on the MMS strategy quickly approaches the current NICE thresholds for cost-effectiveness when extrapolated out to lifetime as compared with the within-trial ICER estimates. Whether MMS could be recommended on economic grounds would depend on the confirmation and size of the mortality benefit at the end of an ongoing follow-up of the UKCTOCS cohort.

Ovarian cancer is the sixth most common cancer in women with approximately 7300 new cases diagnosed in the United Kingdom in 2013 (Cancer Research UK, 2016). Since the late 1970s, incidence has increased by almost 15%. It remains the most common cause of gynaecological cancer death. Whereas <5% of women survive the disease for 5 years if diagnosed at the most advanced stage (IV), when diagnosed early (at stage 1) 90% survive. A successful screening programme would therefore be a highly valued public health-care intervention.

The primary aim of this paper is to draw on the clinical results arising from the mortality reduction seen in the UK Collaborative Trial of Ovarian Cancer Screening (UKCTOCS) to estimate a within-trial period incremental cost-effectiveness ratio (ICER) to assess the value to the UK National Health Service (NHS)-covered population from the initiation of two screening scenarios as compared with a no-screening (control) group in which no screening for ovarian cancer was undertaken (Jacobs *et al*, 2016). The first screening programme was annual multimodal screening (MMS), which is detection of raised serum levels of CA125 using a risk of ovarian cancer algorithm (ROCA) to identify women who may have ovarian cancer (potential cases). The second programme relies on the identification of potential cases through annual transvaginal ultrasound screening (USS). UKCTOCS reported indicative findings that positive mortality benefit might well be gained from an ovarian cancer screening programme. This paper supplements the reported clinical results with estimates of the cost-effectiveness of the MMS and USS screening programmes compared separately with a no-screening arm based on individual patient level trial data.

As well as presenting within-trial ICERs, we also present incremental cost-effectiveness results for the MMS programme *vs* no screening that extrapolates findings past the end of the currently published 14-year follow-up in UKCTOCS. This extrapolation helps to counter the long-lead times required to establish the mortality benefits from an ovarian cancer screening programme. Given the uncertainties involved in extrapolation, we draw upon two different modelling approaches. The first is founded upon direct predictions based on the underlying mortality rates revealed by the trial and associated predicted costs. The second uses a Markov model incorporating the UKCTOCS results to extrapolate costs and effects over a hypothetical population cohort. While necessarily adopting a different model structure, this latter approach has the additional advantage of allowing assessment of competing mortality risks and quality of adjusted life years (QALY).

## Methods

### Patients, setting and comparisons

In the UKCTOCS trial, 202 638 women aged between 50 and 75 years were recruited between 17 April 2001 and 29 September 2005 through 13 UK NHS Trusts. They were randomly allocated between 1 June 2001 and 21 October 2005 to either annual MMS (50 640) using serum CA125 interpreted through an algorithm (ROCA) with transvaginal ultrasound as a second-line test or to annual transvaginal USS (50 639 individuals) or to no screening (101 359 individuals) (Jacobs *et al*, 2016). After exclusions relating to preexisting ovarian cancer or death/loss to follow-up between randomisation and initiation of screening, the total population of women analysed was 202 546, of which 101 299 were in the no-screening arm, 50 624 were in the MMS arm and 50 623 were in the USS arm. Screening was completed at the end of 2011 with follow-up till the end of 2014.

The primary outcome was ovarian cancer death confirmed by an independent outcome review committee by the end of December 2014. Ovarian cancer was defined as malignant neoplasms of the ovary (ICD-10 C56), which included primary non-epithelial ovarian cancer, borderline epithelial ovarian cancer and invasive epithelial ovarian cancer; malignant neoplasms of the fallopian tube (ICD-10 C57.0); and undesignated malignancies of the ovaries, fallopian tube or peritoneum. Primary peritoneal cancer as defined by WHO 2003 was not part of the primary outcome. The trial compared ovarian cancer deaths in the MMS and USS *vs* no-screening groups. Survival time was estimated from the date of randomisation to the date of death due to the primary outcome or censoring (where censoring included death from other causes or loss to follow-up). At the end of the study, 649 (0.32%) women had died of ovarian cancer: 347 (0.34%) in the no-screening arm, 148 (0.29%) in the MMS arm; and 154 (0.30%) in the USS arm. The mortality reduction over the complete follow-up time of 14 years was 15% (95% CI −3 to 30; *P*=0.10) in the MMS arm and 11% (95% CI −7 to 27; *P*=0.21) in the USS arm.

The cumulative hazards for the two screening arms began to separate after 7 years for the MMS *vs* no-screening group and 9 years for the USS *vs* no-screening group comparisons, revealing a substantial delayed effect of screening on mortality. Analysis of this delayed effect showed that screening had a statistically significant impact on mortality when this lead time was accounted for. Over the later trial period, from 7 to 14 years, in the MMS screened group there was a statistically significant mortality reduction of 23% (95% CI 1 to 46). For the USS group, the 7–14-year mortality impact was 21% (95% CI −2 to 42). At censorship, the no-screening group ovarian cancer mortality rate was continuing to rise linearly, whereas the MMS and USS group rates appeared to be plateauing (Jacobs *et al*, 2016).

### Form of evaluation and perspective

In this analysis, we report an incremental cost-effectiveness analysis of the MMS and USS screening programmes separately comparing them to a no-screening arm over the period of the trial. As USS is strictly dominated (is more costly and less effective than) by MMS, the USS *vs* control result is reported for information only for the within-trial analysis. The analysis is based on individual patient-level data collected during the trial and is assessed from the perspective of a national NHS screening programme. We therefore analyse only direct health service costs covering the programme costs of the MMS and USS screening and the subsequent treatment costs.

**Figure 1:**
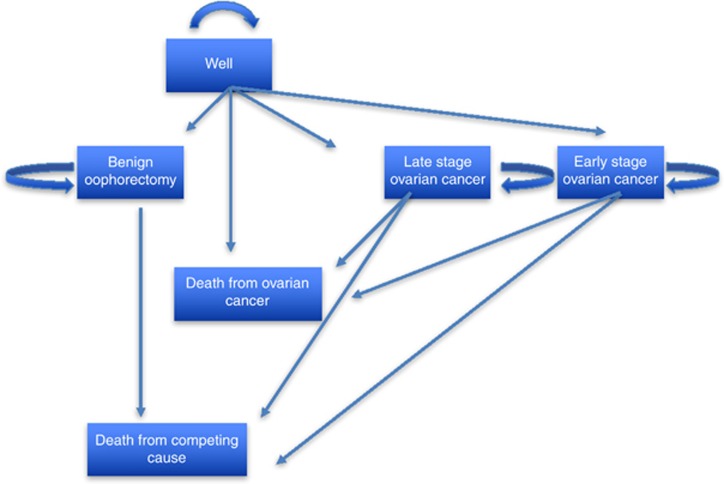
**Markov transition states.**

**Figure 2:**
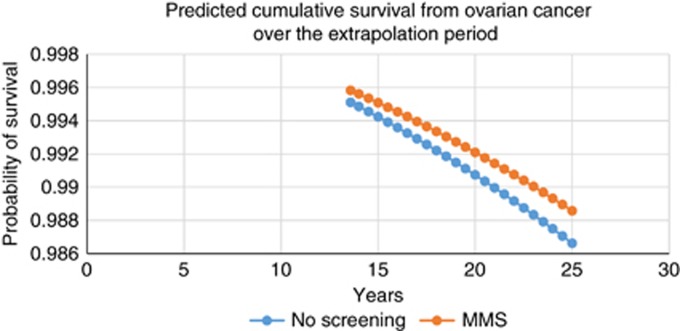
**Predicted cumulative survival probability from ovarian cancer over the extrapolation period for the MMS *vs* no-screening comparison.**

The primary cost-effectiveness analysis therefore relates to assessment of the MMS and USS screening programmes compared with the control population who were not subject to screening within the trial follow-up period of 14 years. As there was a long-lead time required to establish mortality benefit, as represented by the separation of cumulative mortality rates and within trial hazard rates only after 7 years across the screening arms and the control arm of UKCTOCS (Jacobs *et al*, 2016; Figures 1, 2 and), and given that USS was dominated by MMS, we also present a secondary ICER for the MMS group alone compared with the no-screening group estimated over a 25-year period through extrapolating cumulative mortality and costs beyond the end of the trial follow-up period of 14 years. The 25-year period was based on the median age (60.6; IQR 56, 66) of women at randomisation and UK ONS Life Table data estimating life expectancy at this age to be approximately 25 years. Recognising the uncertainty associated with extrapolation, we further undertake Markov modelling of a hypothetical population cohort, based on the UKCTOCS population, to extrapolate the ICER. The Markov model is based on a (‘well’) population of 60-year-old females who transition through states of benign oophorectomy, early-stage ovarian cancer, advanced ovarian cancer, death from ovarian cancer and death from competing mortality. Data taken directly from UKCTOCS provided information on the states of benign oophorectomy, early-stage ovarian cancer, advanced ovarian cancer and death from ovarian cancer, while ONS Life Tables provided the data on competing risks.

### Health outcome

The effectiveness of the screening programme was based directly on the trial primary outcome of mortality due to ovarian cancer, which was converted to life years gained (LYG) for the within-trial analysis. We used the Kaplan–Meier product limit estimates of time to death from ovarian cancer, which were reported in UKCTOCS (Jacobs *et al*, 2016), to calculate the average gain in life expectancy for the MMS and USS screening arms within the trial period as estimated by a calculated restricted mean. The MMS extrapolation, estimated through predicted mortality rates, also reports incremental cost per LYG from the screening programme *vs* no screening, while the Markov model reports QALYs through incorporating secondary data on QALY tariffs. Life years and QALYs gained were discounted at the UK National Institute of Clinical Excellence (NICE) recommended rate of 1.5% per annum for public health interventions as well as at the general recommended rate of 3.5% per annum for health-care treatment programmes (NICE, 2012a, 2012b; NICE, 2013).

### Resource use and cost

For each patient within the trial, all resource usage relative to screening was captured. For those referred for assessment due to screen findings, data related to clinic visits, additional imaging, blood tests, trial related surgery, all chemotherapy agents and the number of cycles recorded for those treated for ovarian cancer and follow-up clinical assessment was captured through review of medical notes. We contacted treating clinicians irrespective of whether they were located in the NHS or private sector to obtain medical notes. The majority of the patients had treatment in the NHS with a small minority treated privately. Only one woman who had undergone trial surgery refused access to notes. Once we mapped resource use, the 2013–2014 NHS tariff prices associated with relevant hospital episodes (in-patient, day case and outpatient), procedures, blood tests and clinics were attached to these visits (Department of Health, 2014). The unit costs arising from treatment of ovarian cancer with chemotherapy agents were supplemented from a number of secondary sources, primarily reports from NICE (UK) and the British National Formulary prices (RICS, 2014).

The only exception to the use of published unit costs was the unit cost of the CA125–ROCA test used to predict the likelihood of ovarian cancer. This test is currently not available in the NHS. The cost a private health care sector CA125 test is approximately £85 (range 75–95), relative to the NHS cost of the CA125 test of £10. The cost of the ROCA as currently performed in the UK private health-care sector is £150. The unit cost of the ROCA test in the NHS was therefore estimated through using a UK private health sector average 8.5-fold mark-up for CA125 over the NHS diagnostic test costs, with a returned estimate of £17 for the combined CA125–ROCA test if it were to be performed in the NHS. An estimate of £20 was therefore used in the base-case analysis and subjected to extensive sensitivity analysis to account for the gross uncertainty surrounding this estimated NHS value. The direct screening costs per patient over the trial period represent approximately 50% of the total individual per patient costs for those in the screening arms.

All unit costs are reported in Table 1. These unit costs, given in 2013/14 prices (£ sterling), were combined with individual patient-specific resource volumes to obtain a total cost per patient for each type of resource and year. These were aggregated to provide a total cost per patient over the entire period of the trial in each arm, which was used to estimate the mean cost per patient screening arm.

Given the presence of right censoring in the cost data, the approach recommended by Lin *et al* (1997) was used to adjust the within-trial cost estimates for censoring. As individual cost history information at intermediate points in time was available, the chosen estimator proposed by Lin *et al* (1997) partitions the entire study period into discrete time intervals of 1 year in our case and makes use of individual cost histories to derive an estimate of average cost within each interval of the partition. The final estimate of average cost over the whole period of analysis is then based on weighting each interval cost estimate by the respective Kaplan–Meier probability of survival to the start of the interval and aggregating these interval cost estimates across the entire analysis period. In this manner, an estimate of mean total cost per patient adjusted for censoring was derived for each of the trial arms.

Combining the above cost and effect estimates to produce differences in the average costs relative to differences in the average effects results in an ICER statistic for each comparison. To address the uncertainty around these values, Fieller’s method for estimating the confidence intervals of ratios was used (Fieller, 1954).

### Extrapolation

Given the age of the trial population at randomisation (median 60.6; IQR 56, 66) and that the evaluation assesses the effects of a screening programme, extrapolating the within-trial results out to 25 years was considered appropriate, as this is the life expectancy of a 60-year-old female in the United Kingdom, in order to capture the delayed effects of the programme while accounting for its future costs.

Given the uncertainty associated with extrapolation, two approaches were adopted. The first is based on predicting future mortality and cost from the trial population data directly. The second is based on Markov modelling of a hypothetical cohort that used the within-trial data to estimate transition probabilities across different states.

For the first extrapolation of effect, the parametric estimates of time to ovarian cancer death by Royston and Parmar (2002), which reported results consistent with the Kaplan–Meier estimates over the trial period (Jacobs *et al*, 2016), were used to predict LYG beyond the end of the trial. This allowed better representation of the full gain in improved mortality from ovarian cancer witnessed as screening was affected by considerable lead time. For the extrapolation the estimates by Royston and Parmar were based on the specification shown to give the best fit to the trial data through application of the Akaike Information Criterion of model selection (Royston and Parmar, 2002). No account was taken of competing risks given that there was no (statistical) difference observed in these risks across the groups within the trial. Analysis was therefore based purely on the trial-estimated ovarian mortality rates. Extrapolated LYGs were discounted at both 1.5% and 3.5%.

To estimate the relevant costs over the period of the extrapolation, the method suggested by Etzioni *et al* (1999) was adopted. Here the extrapolation period is disaggregated into years and for each year the average cost was assumed to consist of two components. The first component gives an estimate of average cost for those who are expected to survive the year based on the within-trial annual observed costs of the survivors and weighted by a parametrically estimated probability of surviving the year. The second component gives an estimate of average cost for those who are expected to die in the year based on the within-trial annual observed costs of those who die and weighted by the respective estimate of the probability of dying in the year. To derive the parametric estimates of survival for each year of the extrapolation period, the estimates by Royston and Parmar were used.

Given the approach by Etzioni *et al* (1999) adopted here, as detailed above, future costs are assumed to follow a similar pattern to that observed within the trial, that is, they assume that patients will continue to receive screening, testing and any necessary treatment beyond the end of the trial and up to the end of the extrapolation period.

The second method of extrapolation was based on a Markov model, with a 1-year cycle, as given in Figure 1, which identifies the transition and terminal states. All data, apart from that on competing risk and quality of life (QoL), values were taken from the UKCTOCS. The transitions from well-to-benign oophorectomy, early-stage ovarian cancer, late-stage ovarian cancer and death from ovarian cancer were based on UKCTOCS within-trial Kaplan–Meier estimates of annual hazards to these end points, which were converted into annual transition probabilities. The QoL data were taken from Edwards *et al* (2015) who reviewed 187 papers reporting QoL data relating to ovarian cancer, of which 27 provided data suitable for use in economic evaluations. Although their concern was with advanced recurrent and refractory ovarian cancer, the study reported QoL tariffs for stable and progressive disease, estimated at 0.718 and 0.649, respectively. These values were based on a sample population of >600 patients and were generally representative of values used for similar states in other studies. We take these values to proxy the QoL tariffs for early-stage ovarian cancer (0.718) and advanced ovarian cancer (0.649) within the Markov model. The competing risk of mortality was based on UK ONS Life Tables of annual mortality rates for 60-year-old women, which when combined with the ovarian cancer mortality rates allowed the cohort to be followed until all had reached death.

For the Markov model, costs were attributed to each state on the following basis. The screening cost was taken as CA125 plus ROCA at £20, plus phlebotomy at £3 and a specialist gynaecological outpatient visit cost of £109. The cost of benign oophorectomy was £2,275 plus an additional outpatient follow-up visit of £139. Early and advanced ovarian cancer was recorded for each of the UKCTOCS participants. In the early ovarian cancer state that includes borderline and non-epithelial ovarian cancer, based on the trial findings an average of 49% of individuals received surgery alone and 51% received surgery and chemotherapy, giving a weighted average cost of £3422, including all surgeries, chemotherapies and outpatient follow-ups; while the advanced ovarian cancer state, where 29% of UKCTOCS individuals had chemotherapy, 8% had surgery alone and 63% had surgery and chemotherapy had a weighted average cost, estimated on a similar basis of £5666.

### Sensitivity analysis

Although the majority of costs reflect NHS treatment costs, as noted above the combined CA125–ROCA unit cost, which is central to the programme cost of the MMS arm, is at present unknown within the NHS as the ROCA is not routinely performed in the public sector. The £20 base-case CA125–ROCA cost was estimated from the known UK private hospital sector cost of the CA125 test and ROCA adjusted through the estimated private-to-public hospital sector mark-up. Given the uncertainty over this unit cost, univariate sensitivity analysis was undertaken with the CA125–ROCA unit cost assessed at £15, £30, £40 and £50 per test, respectively, to assess the impact of this critical value on the ICER estimates. This sensitivity analysis was applied to both the within-trial and the predicted extrapolation analysis. The Markov model applied probabilistic sensitivity analysis to a distribution of CA125–ROCA costs ranging from £15 to £50 using a uniform distribution. Only the control *vs* MMS ICERs are reported for the within-trial sensitivity analysis, the predicted extrapolations and the Markov modelling as the trial USS strategy is completely dominated by the MMS strategy.

### Ethics

Ethical approval was by the UK North West Multicentre Research Ethics Committees (North West MREC 00/8/34).

## Results

We report the ICERs for the within-trial comparisons of the MMS group *vs* no-screening group and the USS group *vs* no-screening group, as well as for the MMS *vs* control extrapolation beyond the trial follow-up period of 14 years out to 25 years, the latter chosen as reflecting a reasonable aggregate survival time when account is taken of competing risks for the trial population. Results are reported with discount rates at 1.5% applied to the effects (LYG and QALYs) and to the costs, as the main results reflect the view that screening is considered a public health intervention, and with LYG and QALYs discounted at 3.5%. We report the within-trial results as mean estimates accompanied by their variance estimators, with ICERs reported together with their 95% confidence intervals using the method by Fieller (1954).

The mean time to death, defined by the primary outcome measure death from ovarian cancer, is reported in Table 2 by screening arm. The results reproduce those reported in the published UKCTOCS analysis and supplement these by the discounted estimates of time to death from ovarian cancer using discount rates of 1.5% and 3.5%. As can be seen, and consistent with the trial results, the Kaplan–Meier estimated time to death from ovarian cancer in the control arm is 13.5574 years undiscounted and 12.36565 years when discounted at 1.5% (11.01755 years when discounted at 3.5%). For the MMS treatment arm, the respective times to death from ovarian cancer are 13.5607 years for undiscounted estimates and 12.3685 years when discounted at 1.5% (11.01979 years discounted at 3.5%), while for the USS arm they are 13.5597 years (undiscounted) and 12.3676 years when discounted at 1.5% (11.0191 years when discounted at 3.5%).

Table 3 reports the mean cost per patient over the duration of the study by programme allocation, discounted at 1.5% and 3.5% per year, adjusted for censoring. As shown, and not surprisingly, the no-screening arm exhibited extremely low costs over the trial period; £135 discounted at 1.5% (£122 discounted at 3.5%) when adjusted for censoring. These costs are essentially a ‘do-nothing’ intervention plus treatment costs arising from the small number, relative to the total population, of individuals in this arm presenting with ovarian cancer at some point within the trial. The MMS group had a mean (adjusted for censoring) cost of £391 when discounted at 1.5%, (£360 discounted at 3.5%) during the trial period. The USS screening arm had a much more expensive mean (adjusted for censoring) cost of £1342 when discounted at 1.5% (£1259 discounted at 3.5%) during the trial period. In all cases, these mean costs represent the screening cost plus any treatment costs for detected ovarian cancer averaged or the relevant population.

These cost and effect estimates formed the basis for the estimation of the within-trial ICERs, which are reported in Table 4. These ICERs are calculated as the incremental cost per LYG for the MMS arm *vs* no-screening arm and the USS arm *vs* no-screening arm. In each case, both costs and LYG are discounted at 1.5% as well as 3.5%. The ICERs account for censoring in both cost and effect estimates. As estimated, the USS *vs* no-screening ICER is extremely high, calculated to be £625 801 (95% CI £620 451, £631 245) per LYG at a 1.5% discount rate and £748 315 (95% CI £741 446, £755 312) per LYG at a 3.5% discount rate. These high values for the ICERs are due to a number of factors. First, the small overall benefit derived in terms of time to death from ovarian cancer from the within trial USS screening programme is a reflection of the long-lead time, of around 7 years, to the associated mortality gain from screening. Coupled with the effect of discounting and the impact of the ICER being a calculated ratio, this gives rise to the high USS ICER estimate.

The estimated within-trial cost per LYG arising from the MMS programme is lower at £91 452 (95% CI £90 909, £92 001) per LYG when discounted at 1.5% and £106 497 (95% CI £105 840, £107 162) per LYG when discounted at 3.5% but remains relatively high for similar reasons to those outlined for the USS *vs* no-screening arm. Following these results and in accordance with the approach adopted in the clinical trial mortality analysis (Jacobs *et al*, 2016), the subsequent discussion of the cost-effectiveness of the screening programme focuses on the comparison of the MMS programme *vs* no-screening arms, given the dominance of MMS over USS.

Given the long-lead time experienced to derive mortality benefit and given that the UKCTOCs trial shows widely divergent hazard rates across the control and MMS population after 7 years (Jacobs *et al*, 2016), we extend the analysis to include an extrapolation of the ICER out to 25 years. This extrapolated period of an additional 11 years is chosen as a reasonable length over which average life expectancy in this age group, given competing risks, might be extended; ONS estimates average life expectancy of a 60-year-old female to be 25.22 years. Figure 2 shows the resultant extrapolated cumulative survival curves for the MMS and no-screening arms, which we believe to be conservative estimates of the health gain from screening given the increasingly divergent hazard rates across the MMS and no-screening groups seen in the trial analysis as documented in Figure 3 in Jacobs *et al*, 2016. The extrapolated ICERs for the MMS *vs* no-screening population discounted at 1.5% and 3.5% are also reported in Table 4 alongside the main within-trial results. The reported ICERs reflect both increasing treatment cost differences over time but, more importantly, increasing LYG as the screening programme matures and are estimated at £30 033 per LYG when a discount rate of 1.5% is applied to both costs and effects and at £35 544 per LYG when discounted at 3.5%.

The results of the univariate sensitivity analysis was limited to the ICER for the MMS *vs* no screening and where the CA125–ROCA unit cost were assumed to be £15, £30, £40 and £50 per test, respectively, are reported in Table 5. These values were deemed representative of the most plausible range of costs that could be negotiated through the NHS for the CA125–ROCA test. A value >£50 for this test was not considered, as the resultant ICER estimate relating to the within-trial analysis reaches £173 258 per LYG and even in the extrapolation period of analysis (for the MMS *vs* no-screening comparison) the ICER is £56 962 per LYG. Further increases in screening cost would merely push these ICERs higher.

In the within-trial sensitivity analysis, the estimated ICERs for the MSS *vs* no-screening comparisons remain high. The average cost per LYG ranges from £77 818 per LYG (Table 5; confidence intervals also reported) when the CA125–ROCA cost was £15 to £173 258 per LYG when the CA125–ROCA test costs was increased to £50 and outcomes are discounted at 1.5% (if a discount rate of 3.5% is applied, the ICER range is £90 549 per LYG with the CA125–ROCA test cost £15 to £202 189 per LYG with CA125–ROCA test cost £50).

Again, these relatively high within-trial ICERs reflect the fact that there is a long-lead time associated with the accrual of the mortality benefits gained from screening. Accounting for these long-lead times through extrapolating the costs and mortality benefits over a 25-year period, the ICERs fall considerably.

In the base-case predicted extrapolation analysis, using the predictions of mortality rates by Royston and Parmar and the method for extrapolating costs by Etzioni *et al* (1999), with the CA125–ROCA test cost estimated at £20 and discounting at 1.5%, the incremental cost per LYG for the MMS intervention was calculated to be £30 033 per LYG compared with no screening (£35 544 per LYG discounting at 3.5%). If the CA125–ROCA test cost falls to £15, the ICER becomes £25 545 per LYG at a 1.5% discount rate (£30 220 per LYG discounting at 3.5%). Over the total 25-year period of analysis (within trial and extrapolation) at a 1.5% per annum discount rate, the ICER rises to £39 009 per LYG with the CA125–ROCA test at £30, £47 986 per LYG with the CA125–ROCA at £40 and £56 962 per LYG with the CA125–ROCA test cost at £50.

For the Markov model, the extrapolated LYG are estimated to be 0.039 when discounted at 1.5% and, although not directly comparable as the model forces individuals through transition states, are greater than the 0.014 LYG estimated through the predictive extrapolation. Given that the Markov model incorporates competing mortality risk, the direction of difference is correct. The extrapolated QALYs in the Markov model are estimated to be 0.0581, as based on the average effect size seen during the UKCTOCS holding across the extrapolation. The base-case analysis in the Markov model comparing the MMS to no screening estimates an ICER, using QALYs as the outcome, to be £46 922 per QALY gained discounting at 1.5% (£54 267 per QALY when both costs and effects are discounted at 3.5%). The probabilistic sensitivity analysis, applying a uniform distribution to a CA125–ROCA cost which lies between £10 and £50, returns a minimum ICER of £45 030 per QALY and a maximum value of £61 134 per QALY.

## Discussion

We report on the cost-effectiveness of screening using within-trial data from one of the world’s largest multicentre randomised controlled trials involving 202 546 women, 673 765(median of 8 per women in screen arms) annual screens and 2.2 million women-years of follow-up. (Jacobs *et al*, 2016). Our analysis suggests that, after accounting for the lead time required to establish full mortality benefits, a national OCS programme based on the MMS strategy when extrapolated out to lifetime could approach the current NICE thresholds for cost-effectiveness (£30 033 per LYG on predictive extrapolation and £46 922 per QALY on Markov modelling) as compared with the within-trial ICER estimates (£91 452–£77 818 per LYG.). This suggests that MMS could be recommended on economic grounds if a definitive mortality benefit of 20% is confirmed on follow-up of the UKCTOCS cohort.

Data on cost-effectiveness of screening for ovarian cancer is limited. The modelling undertaken is limited and based on various assumptions concerning the reliability of screening regimes, with few taking account of QoL considerations. Edwards *et al* (2015) and Sfakianos and Havrileskey (2011) have provided comprehensive reviews of this area, detailing that none have been based on long-term trials. As a result, the evidence base for the cost-effectiveness of screening for ovarian cancer is, to this point, weak.

This analysis is the first to estimate ICERs based directly on within-trial (UKCTOCS) individual patient data. Not surprisingly, given the impact of the long-lead time taken to establish the health benefits, the estimated within-trial ICERs are relatively high. The most favourable within-trial ratio relating to the comparison of the MMS arm *vs* no screening is calculated to be £91 452 per LYG accounting for censoring in both cost and mortality estimates and applying the NICE public health programme discount rate of 1.5% per annum. It is important to note that this relatively high cost-effectiveness ratio is not a result of the average cost per patient being large over the trial period but a direct consequence of the trial not being long enough to adequately reflect the full mortality benefits likely to be gained from the MMS programme.

As a result, the extrapolation of the within-trial results becomes critical. Extrapolation highlights the problems of model uncertainty, as well as the general issues surrounding prediction. We have therefore resorted to two extrapolations: one based on predictions of within-trial mortality rates and treatment costs beyond the trial period and one based on the development of a Markov model founded on data from the UKCTOCS trial applied to a hypothetical cohort moved through a number of health states.

One advantage of the Markov modelling is that it can incorporate QoL data gained from outside the trial. Although extensive QoL data were collected during the trial, these were related to screening or profile data and are not readily convertible to the tariff values required to calculate QALY gains (Barrett *et al*, 2014). The calculation of QALYs would have, for example, allowed precise quantitative estimation of the impact that screening complications and false-positive results had on the QoL of the women involved. Such an impact, we believe would have been small as <1% of the women in either the USS or MMS screening arms experienced screen complications, all of which were mild such as bruising or discomfort during scanning/blood test, while the number of false-positive surgeries performed were 50 per 10 000 screens in the USS group and 14 per 10 000 screens in the MMS group. The costs of these false-positive surgeries are captured in all the reported analysis, but it is only in the Markov analysis that QALY estimates were provided.

The predictive modelling of the ICER provides an estimate for the MMS programme relative to the no-screening population extrapolated out to a 25-year period that falls dramatically to £30 033 per LYG when a discount rate of 1.5% is applied (£35 544 per LYG when a discount rate of 3.5% is applied), while for the Markov model the extrapolated cost per QALY, in an analysis that also incorporates competing mortality risks, is estimated to be £46 922 per QALY.

Both provide indicative evidence that, if using the conventional NICE threshold of £20 000 per QALY to £30 000 per QALY to deem interventions cost-effective, a screening strategy based on the MMS programme fast approaches becoming cost-effective when compared with the within-trial analysis when the programme is lengthened using within-trial mortality benefits as the basis of extrapolation.

The long-lead time associated with establishing mortality benefit from ovarian cancer screening has meant that the full benefits to be realised from such a programme have not been established authoritatively. This is further complicated by the presence of preexisting cancers within the trial population at the first screen. Indeed, in a prespecified secondary analysis that excluded prevalent cases from the analysis population a statistically significant 28% mortality reduction was shown in the MMS arm after 7 years of the trial (Jacobs *et al*, 2016). If this increased mortality gain were to convert into a larger gain in time to survival for the MMS arm, this would greatly improve the cost-effectiveness estimates given that we are using extremely low values in the denominator of the calculated ICERs. Given that the trial population is a mixed cohort of prevalence and incidence cases, more complex modelling than undertaken here would be required to fully establish this case.

The findings are not only highly dependent on the average effect size of the mortality reduction associated with screening but also the cost of the CA125–ROCA test, assuming this test is adopted by the NHS. The base-case analysis of the predictive extrapolation model and of the Markov model both use a CA125–ROCA cost of £20. Even this seems relatively high as the current average NHS cost for histopathology and histology tests within the NHS are £10. Moreover, if as is possible given the continuing divergence of the hazard rates for the no-screening and MMS arms at censorship for mortality analysis, the screening effect benefit continues to grow, the ICER will continue to approach the NICE threshold. We have been deliberately conservative, given the uncertainties associated with extrapolation and prediction and the difficulties of estimating the cost to the NHS of the CA125–ROCA test.

In conclusion, the results of the extrapolation over lifetime suggest that a public health programme of screening for ovarian cancer could become cost-effective within an NHS setting if the mortality benefit from screening continues to increase over time. Any definitive conclusion as to whether MMS could be recommended on economic grounds would depend on the confirmation and size of the mortality benefit at the end of ongoing follow-up of the UKCTOCS cohort.

## Figures and Tables

**Table 1:** Unit costs applied to the incremental cost-effectiveness ratios

**NHS hospital tariffs 2013–2014**			
**Operation**	**In-patient**	**Day case**	**Outpatient**
Laparotomy	£2275	£569	£139
Operative laparoscopy	£4852	£2075	£139
Hysteroscopy/D&C	£1284	£898	£205
Diagnostic laparoscopy	£2239	£1579	£205
Diagnostic laparoscopy/laparotomy	£3523	£2477	£205
Vaginal surgery	£1207	£484	£205
Hysterectgomy	£3703	£1893	£156
Oophorectomy	£2275	£569	£139
Omentectomy	£2275	£569	£139
**Excision**			
Colon	£884	£590	£139
Bowel	£5478	£565	£139
Appendectomy	£3144	£1709	£139
Rectum	£1461	£1040	£139
Spleen	£3951	£1413	£139
Hormone replacement therapy (with oophorectomy)	£2275	£569	
Transvaginal ultrasound			£150

**Unit costs for tests and clinics 2013–2014**	
Cytology	£8
Histopathology and histology	£10
Intergrated blood services	£2
Clinical biochemistry	£1
Haematology	£3
Immunology	£5
Microbiology	£7
Phlebotomy	£3
Local gynaecological cancer MDT meetings	£18
Specialist gynaecological cancer MDT meetings	£109
Estimated CA125+ROCA cost	£20

Abbreviations: CA125=cancer antigen 125; D&C=dilation and curettage; MDT=multidisciplinary team; NHS=National Health Service; ROCA=risk of ovarian cancer algorithm.

**Table 2:** Mean time to primary death by treatment group within the trial period

	**Control (*****N*****=101 299)**	**Multimodal (*****N*****=50 624)**	**Ultrasound (*****N*****=50 623)**
	**Mean (s.e.)**		
Kaplan–Meier (undiscounted)	13.55736 (0.0014)	13.56069 (0.0019)	13.55967 (0.0019)
Kaplan–Meier (discounted at 1.5%)	12.36565 (0.0012)	12.36845 (0.0017)	12.36758 (0.0017)
Kaplan–Meier (discounted at 3.5%)	11.01755 (0.0011)	11.01979 (0.0014)	11.01907 (0.0014)

**Table 3:** Mean cost per patient over the trial period (ROCA=£20)

	**Control**		**Multimodal**		**Ultrasound**	
	**Mean**^a^	**Variance**^a^	**Mean**^a^	**Variance**^a^	**Mean**^a^	**Variance**^a^
Adjusted for censoring, undiscounted	£146	£40	£416	£56	£1412	£77
Adjusted for censoring, discounted at 1.5%	£135	£30	£391	£47	£1342	£66
Adjusted for censoring, discounted at 3.5%	£122	£22	£360	£38	£1259	£56

Abbreviation: ROCA=risk of ovarian cancer algorithm.

a Means and variances were estimated using the estimator by Lin *et al* (1997).

**Table 4:** Incremental cost-effectiveness ratios for the MMS *vs* control and USS *vs* control comparisons within the trial period (ROCA=£20)

	**Multimodal** ***vs*** **control**			**Ultrasound** ***vs*** **control**		
	**Cost difference**	**Effect difference**	**ICER (95% confidence interval)**	**Cost difference**	**Effect difference**	**ICER (95% confidence interval)**
Discounted at 1.5% and both costs and effects adjusted for censoring	£256	0.0028	£91 452 per LYG (£90 909, £92 001)	£1208	0.00193	£625 801 per LYG (£620 451, £631 245)
Discounted at 3.5% and both costs and effects adjusted for censoring	£239	0.00224	£106 497 per LYG (£105 840, £107 162)	£1137	0.00152	£748 315 per LYG (£741 446, £755 312)
**Results for multimodal** ***vs*** **control ICER extrapolated to 25 years**						
Discounted at 1.5%	£427	0.01421	£30 033 per LYG			
Discounted at 3.5%	£358	0.01008	£35 544 per LYG			

Abbreviations: ICER=incremental cost-effectiveness ratio; LYG=life year gained; MMS=multimodal screening; ROCA=risk of ovarian cancer algorithm; USS=ultrasound screening. Note: ICER values differ from straight division of cost difference by effect difference due to rounding.

**Table 5:** Incremental cost-effectiveness ratios for the MMS *vs* control and USS *vs* control comparisons within the trial period (ROCA=£15)

	**Multimodal** ***vs*** **control**			**Ultrasound** ***vs*** **control**		
	**Cost difference**	**Effect difference**	**ICER (95% confidence interval)**	**Cost difference**	**Effect difference**	**ICER (95% confidence interval)**
Discounted at 1.5%	£218	0.0028	£77 818 per LYG (£77 356, £78 285)	£1207	0.00193	£625 300 per LYG (£619 954, £630 740)
Discounted at 3.5%	£203	0.00224	£90 549 per LYG (£89 990, £91 115)	£1137	0.00152	£747 717 per LYG (£740 854, £754 708)
**Results for multimodal** ***vs*** **control ICER extrapolated to 25 years (ROCA=£15)**						
Discounted at 1.5%	£363	0.01421	£25 545 per LYG			
Discounted at 3.5%	£305	0.01008	£30 220 per LYG			
**(ROCA=£30)**						
Discounted at 1.5%	£332	0.0028	£118 721 per LYG (£118 017, £119 432)	£1210	0.00193	£626 802 per LYG (621 443, £632 255)
Discounted at 3.5%	£310	0.00224	£138 395 per LYG (£137 542, £139 258)	£1139	0.00152	£749 510 per LYG (£742 630, £756 518)
**Results for multimodal** ***vs*** **control ICER extrapolated to 25 years (ROCA=£30)**						
Discounted at 1.5%	£554	0.01421	£39 009 per LYG			
Discounted at 3.5%	£466	0.01008	£46 194 per LYG			
**(ROCA=£40)**						
Discounted at 1.5%	£409	0.0028	£145 989 per LYG (£145 125, £146 864)	£1212	0.00193	£627 804 per LYG (£622 436, £633 265)
Discounted at 3.5%	£381	0.00224	£170 292 per LYG (£169 243, £171 354)	£1141	0.00152	£750 705 per LYG (£743 814, £757 724)
**Results for multimodal** ***vs*** **control ICER extrapolated to 25 years (ROCA=£40)**						
Discounted at 1.5%	£682	0.01421	£47 986 per LYG			
Discounted at 3.5%	£573	0.01008	£56 843 per LYG			
**(ROCA=£50)**						
Discounted at 1.5%	£485	0.0028	£173 258 per LYG (£172 233, £174 296)	£1214	0.00193	£628 805 per LYG (£623 428, £634 275)
Discounted at 3.5%	£453	0.00224	£202 189 per LYG (£200 945, £203 449)	£1143	0.00152	£751 900 per LYG (£744 998, £758 930)
**Results for multimodal** ***vs*** **control ICER extrapolated to 25 years (ROCA=£50)**						
Discounted at 1.5%	£809	0.01421	£56 962 per LYG			
Discounted at 3.5%	£680	0.01008	£67 492 per LYG			

Abbreviations: ICER=incremental cost-effectiveness ratio; LYG=life year gained; MMS=multimodal screening; ROCA=risk of ovarian cancer algorithm; USS=ultrasound screening. Note: ICER values differ from straight division of cost difference by effect difference due to rounding.
